# Simplified method of plantigrafia for assessing the feet of diabetic patients

**DOI:** 10.1186/1758-5996-7-S1-A248

**Published:** 2015-11-11

**Authors:** Beatriz Bertolaccini Martínez, Silvia Mara Tasso, Adriana Tereza da Silva, Márcio Emílio Cruz Vono Azevedo, Roberto Ribeiro Rocha, Marcos Mesquita Filho

**Affiliations:** 1 Universidade do Vale do Sapucaí, Pouso Alegre, Brazil

## Background

The complications in the feet of diabetic patients are an important cause of morbidity and generate high economic cost to health systems. This includes high rates of amputations and hospitalizations and leads to reduction of work capacity of people still of working age. This also alters the quality of life of these patients. The wounds on the feet reach approximately 15% of patients with diabetes mellitus (DM) throughout life in Brazil. High plantar pressure is a proven risk factor for ulceration among individuals with DM. The photopodoscopia is one of the tools used in screening for high plantar pressure among these subjects. However, an examination photopodoscopy is not accurately demonstrates the high pressure area and there is no specific computer program to analyze the image plant.

## Objectives

Developed a simplified method of plantigrafia for assessing the feet of diabetic patients and a computer program to analyze footprint of diabetic patients.

## Materials and methods

The method was developed by medical professionals and systems analysts from the University of Vale do Sapucai, Minas Gerais, Brazil. It is in registration process with the National Institute of Intellectual Property (INPI). Footprints were taken from 113 subjects using the photopodoscopia and plantigrafia. It was compare high pressure points plant between the two tests. It was analyzed the agreement and intra-rater reliability.

## Results

All patients were type 2 diabetics and 56,6% were women. The average age was 62,8±9,8 yrs. The weighted kappa coefficient was high concordance (Kw>0.79) for the intra-examiner analyses for most of the points studied on both feet.

## Conclusion

The plantigrafia with a specific computer program to analyze the footprints feature ease of handling and low cost, it may represent an important social impact.

